# Insects with 100 million-year-old dinosaur feathers are not ectoparasites

**DOI:** 10.1038/s41467-021-21751-x

**Published:** 2021-03-05

**Authors:** David A. Grimaldi, Isabelle M. Vea

**Affiliations:** 1 Division of Invertebrate Zoology, American Museum of Natural History, New York, NY USA; 2 Department of Biological Sciences, University of Illinois at Chicago, Chicago, IL USA

**Keywords:** Palaeontology, Entomology

**Arising from** Taiping Gao et al. *Nature Communications* 10.1038/s41467-019-13516-4 (2019)

A recent article^1^ describes minute insects that are on or close to two feathers in two pieces of mid-Cretaceous (100 Ma) amber from Myanmar, and states that evidence “strongly suggest[s] that *Mesophthirus* is ectoparasitic”. The feathers are presumably from stem-group, feathered avialan dinosaurs. Despite the abundance of feathers in Burmese amber^2^ and even the rare occurrence of nestling pennaraptorans^2,3^, ticks have been the only ectoparasites found thus far in Burmese amber^4^.

The authors left *Mesophthirus* as *incertae sedis* to any insect order because of what they proposed as an unusual combination of features. We independently concluded that these insects are actually early instar nymphal scale insects (order Hemiptera; suborder Sternorrhyncha; superfamily Coccoidea). As such, *Mesophthirus* could not have been parasitic; their proximity to feathers is a fossilized coincidence unrelated to diet.

Like all of their sternorrhynchan relatives (e.g., aphids, whiteflies, and plant lice), coccoids feed on plant vascular fluids^5^. There are 3–4 nymphal stages (all wingless), the first two of which are active, called “crawlers”. Females are paedomorphic, retaining the wingless condition and other reduced features of the nymphs. Coccoids are abundant and diverse in Cretaceous and Cenozoic ambers around the world^6–11^, particularly the small, winged males, which are easily wafted by air currents. The minute crawlers are also preserved^12,13^ but tend to be overlooked. In one study, crawlers comprised 33% of all coccoids in Baltic amber^12^. Adult females are rarely preserved, a notable exception being the family Ortheziidae, in which adult females have well-developed legs and are quite mobile^10^.

There are several highly distinctive features of *Mesophthirus* that indicate they are coccoid crawlers: very small size (smallest ones ca. 150 μm); body oval to oblong, with no constriction between head, thorax, and abdomen; eyes greatly reduced to one large facet; antenna with only 5–6 antennomeres, apical one largest and having at its apex several long setae and one or shorter, thicker ones; tarsomeres reduced to one (not including the pretarsus) (in *Mesophthirus* two tarsomeres are reported, which is incorrect).

Regarding the apparent clasping of barbules by one specimen of *Mesophthirus*, and barb shaft by another (Fig. 1g, h in ref. ^1^): a large, single pretarsal claw occurs only in anopluran (sucking) lice and three families of mammal-feeding chewing lice, but not in lineages from the basal nodes in anopluran phylogeny. In lice a single claw is strongly curved and folds like a jackknife against an enlarged tarsus, to grasp onto a barbule or hair. The tarsi and pretarsi of *Mesophthirus* show no such specialization, but rather are identical to those of coccoids^5^. Also, specimen CNU-MA2016006 has three legs straddling (not grasping) the feather barbules, the right midleg is partly folded around a barbule. Half of the ten insects are not on a feather. Moreover, the pretarsal claw of *Mesophthirus* is flanked by a pair of long setae clubbed at the tip, which is called tarsal digitules in coccoids^14^. The digitules and large eye facets are especially visible in high-resolution images of paratype specimen CNU-MA2016005, provided to us by the authors along with images of other specimens.

Other features that are consistent with, though hardly restricted to, coccoid crawlers are the following: wingless, dorsoventrally flattened, thorax well developed, with large prothorax (features common throughout nymphal hemimetabolan insects); the last antennomere with “irregular crinkling” (common in paraneopterans, to which Hemiptera, barklice, true lice, and thrips belong); legs short and stout. Abdominal spiracles occur in archaeococcoid nymphs, but the structures described as spiracles by the authors appear to be small cuticular plates bearing minute setae, which are commonly seen in a serial row on each lateral margin of coccoids^14^.

Features of *Mesophthirus* that would seemingly contradict placement in Coccoidea—indeed, in any paraneopteran group—are what are reported to be large chewing mandibles and the presence of palps. In fact, we feel the authors have misinterpreted these structures in the fossils.

The “mandibles” depicted in the interpretive drawing of the holotype of *Mesophthirus engeli*, CNU-MA2016009, are not at all apparent in the photomicrographs of this specimen (Figs. 2a–c in ref. ^1^—reprinted here as Fig. 1d–f). This area actually conforms to the swollen, muscular clypeus, its margins nicely delineated in Figs. 2d, f of ref. ^1^ (but only partly drawn). In Fig. 3c of ref. ^1^ (Fig. 1g here), the thick clypeus is very well imaged, with a slight central furrow, creating a pair of labia-like structures that look like mandibles. There is no evidence in any of the published or high-res photomicrographs provided to us of palps and mandibles. The small central appendage on the posterior margin of the clypeus, visible in the photomicrographs and in their rendering (Figs. 2d–f in ref. ^1^) may be the labium, but this is not certain. The labium is an open tube in coccoids that partly sheaths the long, thread-like stylets; it is quite variable in size (sometimes just a tiny lobe), and always lies between or slightly behind the first pair of coxae, sometimes even between the midcoxae (Fig. 1a, b).

**Fig. 1:**
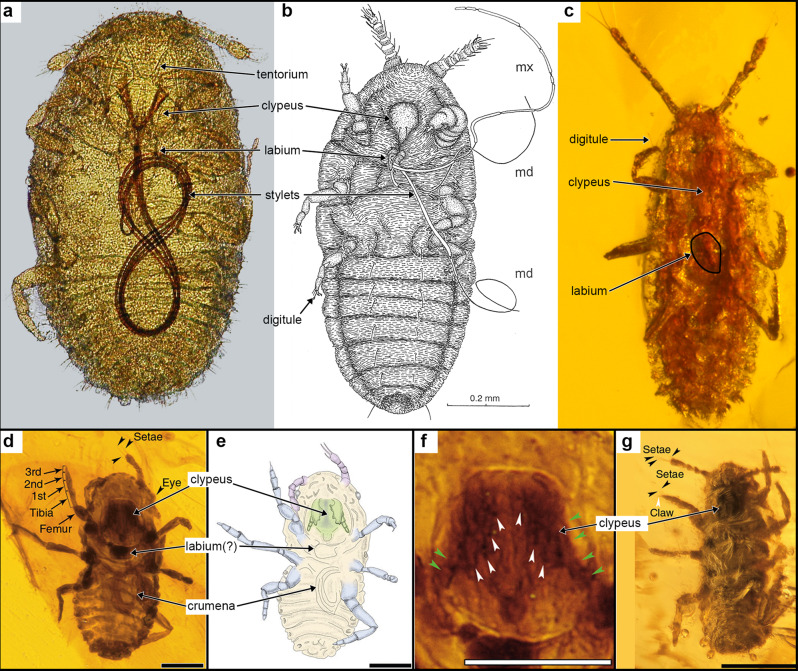
Early instar nymphal Coccoidea, both recent and fossil. Images **d**–**g** are from the original report by Gao et al. (ref. ^1^). **a** Crawler of the extant species *Marchalina hellenica* (Margarodidae), ventral view, showing the internal, figure-8 coiled feeding stylets (the crumena) through the translucent body wall. From ref. ^16^, the crumena is commonly a single coil. **b** Early instar nymph of pityococcid in Baltic amber (ventral view), from ref. ^14^ (original drawing by late Jan Koteja). The feeding styles are fully everted. **c** Early instar nymph, family undetermined, in Burmese amber, AMNH Bu-1327. Ventral view, slightly oblique laterally (making nymph appear slightly thinner than actual). Location of the labium is outlined. Stylets are not everted, and crumena not visible through the opaque body wall. Figures **d**–**g**: From Gao et al. (2019, ref. ^1^). The labels outlined in white are ours; all others are original. Permissions: **a**, ©2006 International Bee Research Association, reprinted by permission of Taylor & Francis Ltd., http://www.tandfonline.com on behalf of ©2006 International Bee Research Association. **b**, courtesy of Dr. Paweł Koteja.

Coccoid stylets—highly modified mandibles and maxillae—are inserted into plant vascular tissue to siphon out fluids for feeding, with the labium acting as a brace at the base. When the stylets are not everted (e.g., Fig. 1b) they are retracted into a long coil within the body, called the crumena (Fig. 1a). A crumena was even rendered in their interpretive drawing of holotype specimen CNU-MA2016009 (Fig. 2b in ref. ^1^, here as Fig. 1e), though it was not labeled as such nor discussed. It is even apparent in photomicrographs of specimens where the cuticle and body are likewise partly transparent (i.e., Figs. 2d, g, in ref. ^1^; here as Fig. 1d). This highly specialized condition of mouthparts is called opisthognathous—where the ventrally situated mouthparts point backward (not hypognathous as the authors mentioned), typical of all thrips (Thysanoptera) and Hemiptera. The mouthparts of *Mesophthirus* are definitely not held forward (prognathous), as in all psocodeans including lice.

The unambiguous morphological evidence, thus, includes the distinctive body shape; the segmentation, setation, and structure of the antennae and tarsi; the large clypeus and coiled feeding stylets in the body, as well as an apparent (but very short) labium. What are reported to be chewing mandibles and palps are not visible to us. These features compel us to place the early instar nymphs in an undetermined family within the Coccoidea. Crawlers in the more diverse families of coccoids (the Neococcoidea) typically have a small pair of lobes bearing a few long setae on the posterior end, but *Mesophthirus* lacks these, so this genus is probably in a family at the base of the Coccoidea phylogeny^14^, probably Ortheziidae based on eye and antennal structure^10^.

How do we explain the presence of the coccoids on the feathers and the putative feeding traces? This is damage that could have been made by anything. If the feathers had shed on the ground or in a nest they could have been consumed by larval dermestid beetles, which today are ubiquitous in and around bird and mammal nests feeding on keratinous debris, and which we know occurred at least by this time period^4^.

Coccoid crawlers are aptly named. They clamber over the surfaces of plants, leaf litter, tree trunks, and no doubt even fallen feathers. Although not coccoids, the closely related adelgid aphids (which also have small nymphs and sessile females) can be transported in the plumage of birds^15^. Coccoids are never ectoparasitic, since the fine mouthparts could never penetrate and draw up tough, dry keratin. Given the abundance of coccoids generally in amber deposits around the world and the abundance of feathers specifically in Burmese amber, a coincidental co-occurrence of the two is quite probable.

## Reporting summary

Further information on research design is available in the Nature Research Reporting Summary linked to this article.

## Supplementary information

Reporting Summary

## Data Availability

The findings of this study are based on the published images cited herein. The additional specimen microphotographed in this paper is deposited in the American Museum of Natural History, specimen number: AMNH Bu-1327.
