# Supplementary material for: Insects with 100 million-year-old dinosaur feathers are not ectoparasites
Source: Nat Commun. 2021 Mar 5;12:1469. doi: 10.1038/s41467-021-21751-x (PMC7935990; doi:10.1038/s41467-021-21751-x)
Supplement: Supplementary file 1 — Reporting Summary [file 41467_2021_21751_MOESM1_ESM.pdf]

nature research

Corresponding author(s): David A. Grimaldi

Last updated by author(s): Jan 28, 2020

Reporting Summary

Nature Research wishes to improve the reproducibility of the work that we publish. This form provides structure for consistency and transparency in reporting. For further information on Nature Research policies, see Authors & Referees and the Editorial Policy Checklist .

Statistics

For all statistical analyses, confirm that the following items are present in the figure legend, table legend, main text, or Methods section.

n/a	Confirmed	
☒	☐	The exact sample size (*n*) for each experimental group/condition, given as a discrete number and unit of measurement
☒	☐	A statement on whether measurements were taken from distinct samples or whether the same sample was measured repeatedly
☒	☐	The statistical test(s) used AND whether they are one- or two-sided<br>*Only common tests should be described solely by name; describe more complex techniques in the Methods section.*
☒	☐	A description of all covariates tested
☒	☐	A description of any assumptions or corrections, such as tests of normality and adjustment for multiple comparisons
☒	☐	A full description of the statistical parameters including central tendency (e.g. means) or other basic estimates (e.g. regression coefficient) AND variation (e.g. standard deviation) or associated estimates of uncertainty (e.g. confidence intervals)
☒	☐	For null hypothesis testing, the test statistic (e.g. *F*, *t*, *r*) with confidence intervals, effect sizes, degrees of freedom and *P* value noted<br>*Give P values as exact values whenever suitable.*
☒	☐	For Bayesian analysis, information on the choice of priors and Markov chain Monte Carlo settings
☒	☐	For hierarchical and complex designs, identification of the appropriate level for tests and full reporting of outcomes
☒	☐	Estimates of effect sizes (e.g. Cohen's *d*, Pearson's *r*), indicating how they were calculated

Our web collection on statistics for biologists contains articles on many of the points above.

Software and code

Policy information about availability of computer code

Data collection	no software used
Data analysis	no software used

For manuscripts utilizing custom algorithms or software that are central to the research but not yet described in published literature, software must be made available to editors/reviewers. We strongly encourage code deposition in a community repository (e.g. GitHub). See the Nature Research guidelines for submitting code & software for further information.

Data

Policy information about availability of data

All manuscripts must include a data availability statement. This statement should provide the following information, where applicable:

- Accession codes, unique identifiers, or web links for publicly available datasets
- A list of figures that have associated raw data
- A description of any restrictions on data availability

The data ultimately resides with specimens, for which museum registration numbers are provided in paper.

Field-specific reporting

Please select the one below that is the best fit for your research. If you are not sure, read the appropriate sections before making your selection.

☐ Life sciences ☐ Behavioural & social sciences ☒ Ecological, evolutionary & environmental sciences

For a reference copy of the document with all sections, see nature.com/documents/nr-reporting-summary-flat.pdf

Ecological, evolutionary & environmental sciences study design

All studies must disclose on these points even when the disclosure is negative.

Study description: A Commentary that provides a redescription and reinterpretation of "unusual" insects that were preserved with 100 million-year old dinosaur feathers. The insects are immature scale insects, which is a plant-feeding group, and so were not feeding on feathers.

Research sample: In this study, DAG and IMV selected and photographed an amber inclusion of scale insect (Coccoidea) crawler from the same deposit (Burmese amber) as the paper being discussed. This specimen was selected for its good preservation and orientation, which enabled to directly compare and discuss its morphology with the Figure of the original paper.

Sampling strategy: This study did not involved quantitative data analysis so data sampling is not applicable here

Data collection: The amber piece was mounted on a slide with glycerin on which a coverslip was added for observation and microphotographing. Microphotography was carried out using a Nikon SMZ1500 microscope with NIC Elements imaging software.

Timing and spatial scale: This study did not use time and spatial data, so such scale is not applicable

Data exclusions: This study is not based on quantitative data analysis so data exclusion is not applicable

Reproducibility: This study is not based on quantitative data analysis so reproducibility is not applicable

Randomization: This study is not based on quantitative data analysis so randomization is not applicable

Blinding: This study is not based on quantitative data analysis so blinding is not applicable

Did the study involve field work? ☐ Yes ☒ No

Reporting for specific materials, systems and methods

We require information from authors about some types of materials, experimental systems and methods used in many studies. Here, indicate whether each material, system or method listed is relevant to your study. If you are not sure if a list item applies to your research, read the appropriate section before selecting a response.

Materials & experimental systems

n/a	Involved in the study	
☒	☐	Antibodies
☒	☐	Eukaryotic cell lines
☐	☒	Palaeontology
☒	☐	Animals and other organisms
☒	☐	Human research participants
☒	☐	Clinical data

Methods

n/a	Involved in the study	
☒	☐	ChIP-seq
☒	☐	Flow cytometry
☒	☐	MRI-based neuroimaging

Palaeontology

Specimen provenance: mid-Cretaceous amber from northern Myanmar, the piece containing AMNH Bu-1327 was purchased in 2000 from Leeward Capital Corporation

Specimen deposition: Specimens reported in the original study that we critiqued reside in the collection of Capital Normal University, Beijing China; in our commentary we include an additional specimen from the American Museum of Natural History in NY, and refer to specimens from other publications (all deposited in other museums).

Dating methods: Dates are cited as per the most widely cited dating study on this amber by Shi et al. (of which D. Grimaldi is a coauthor).

☒ Tick this box to confirm that the raw and calibrated dates are available in the paper or in Supplementary Information.
